# Supplementary material for: Longitudinal Follow-Up of Blood Telomere Length in HIV-Exposed Uninfected Children Having Received One Year of Lopinavir/Ritonavir or Lamivudine as Prophylaxis
Source: Children (Basel). 2021 Sep 10;8(9):796. doi: 10.3390/children8090796 (PMC8468502; doi:10.3390/children8090796)
Supplement: Supplementary file 1 [file children-08-00796-s001.zip › children-1331941-supplementary.pdf]

Supplementary Material

1 Supplementary Data

1.1 Patients and Methods: Telomere Length Assay

Quantitative polymerase chain reaction efficiency, intra- and interplate coefficients of variation

Inter- and intra-plate coefficients of variation were calculated using Ct (n= 3 per plate) obtained after the DNA amplification of telomere and *RPLP0* regions of a controlled DNA from the human embryonic kidney 293T cell line.

Intra-plate coefficient of variation of telomere length, defined by $\Delta TL/TL_{intra}$, was calculated for each plate with the following formula:

$$\Delta TL/TL_{intra} = \frac{Standard\ deviation\ \mathrm{Ct}(Telomere)}{mean\ \mathrm{Ct}(Telomere)} + \frac{Standard\ deviation\ \mathrm{Ct}(RPLP0)}{mean\ \mathrm{Ct}(RPLP0)}$$

$\Delta TL/TL_{intra}$ varied from 0.6% to 5.3%.

Inter-plate coefficient of variation, defined by $\Delta TL/TL_{inter}$, was calculated with the mean and the standard deviation of all $\Delta TL/TL_{intra.}$ $\Delta TL/TL_{inter}$ was 4.03%.

Quality control of quantitative polymerase chain reaction and outliers identification

Twenty-one quantitative polymerase chain reaction plates were necessary to quantify telomere length of the full children population (n = 238, Supplementary Figure S1). As a quality control, all plates with a quantitative polymerase chain reaction efficiency outside the range of the mean of efficiency of all plates ± 2 standard deviation (SD) were repeated. Efficiency for telomere had a mean of 70.8% ± 7.6 and efficiency for *RPLP0* had a mean of 107.0% ± 4.4. We also checked for nuclear DNA degradation by removing samples with a cycle threshold (Ct) for *RPLP0* not comprised between the mean of all the *RPLP0* Ct ± 2SD. Samples with a Ct for telomere not comprised between the mean of all the telomere Ct ± 2SD was considered as outliers and removed from the analysis. The flow chart of the study and the quality control assessment are depicted in Supplementary Figure S1.

1.2 Results: Quality Control of DNA and Outliers Identification

All quantitative polymerase chain reaction plates fulfilled the quality control rule. In the PrEP group, thirty-one CHEU failed the quality control after checking for *RPLP0* and Tel DNA degradation at day-7, week-50, and year-6. In the sub-analysis, two CHEU were also removed after checking for DNA degradation at week-6 and week-50.

2 Supplementary Figures and Tables

2.1 Supplementary Figures

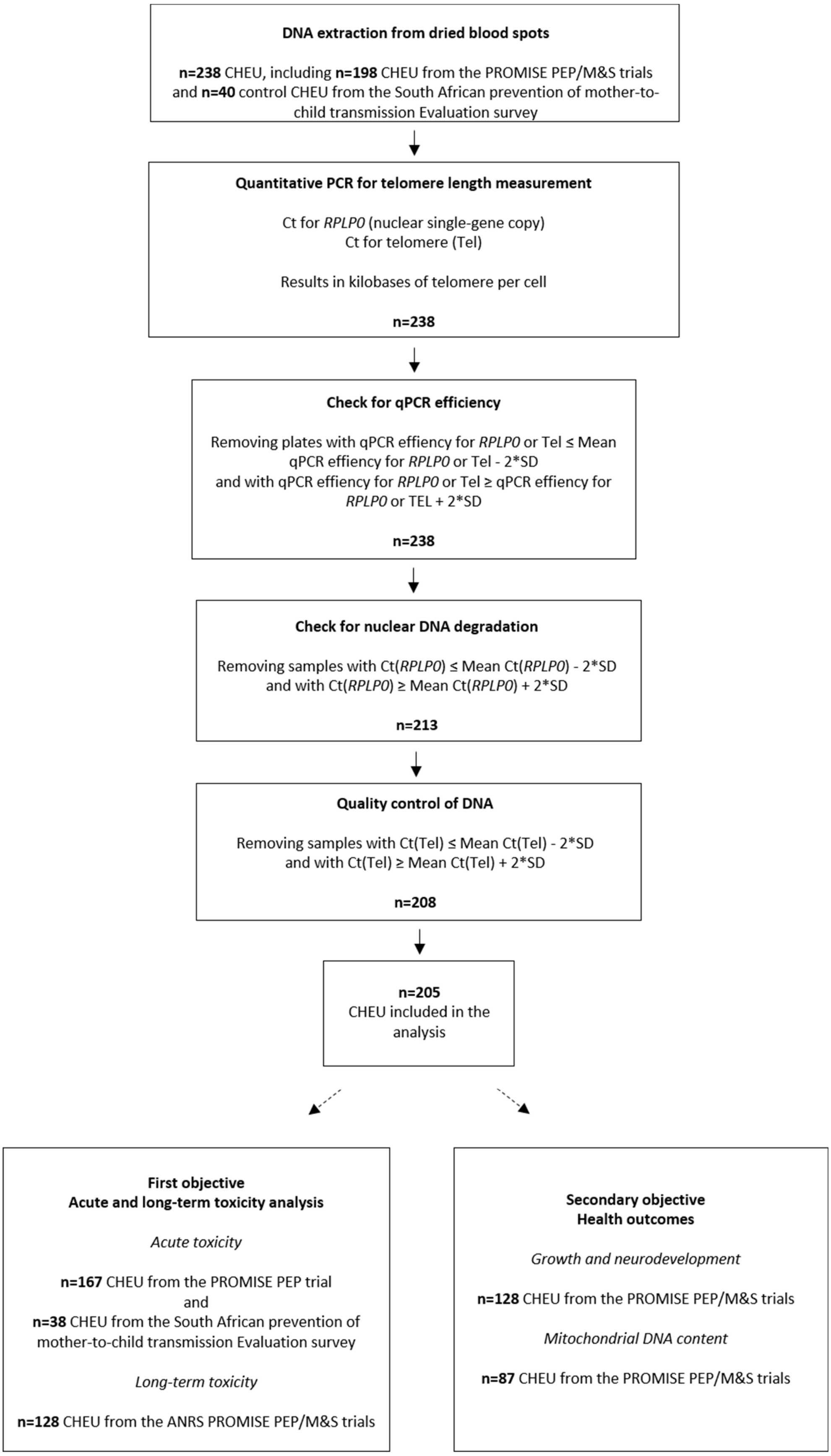

Figure S1. Analysis flow for telomere length measurement. Abbreviations: CHEU, children who are HIV-exposed uninfected; PCR, polymerase chain reaction; qPCR, quantitative PCR; Ct, cycle threshold.

2.2 Supplementary Tables

Supplementary Table S1. Oligomers and primers used for telomere length measurement by quantitative polymerase chain reaction.

		**Sequence**	**Amplicon size**
**Standards (synthetic oligomers)**	Telomere	$(TTAGGG)_{14}$	84pb
	RPLP0	CAGCAAGTGGGAAGGTGTAATCCGTCT CCACAGACAAGGCCAGGACTCGTTTGT ACCCGTTGATGATAGAATGGG	75pb
**Primers**	Forward Telomere	CGGTTTGTTTGGGTTTGGGTTTGGGTTT GGGTTTGGGTT	>76pb
	Reverse Telomere	GGCTTGCCTTACCCTTACCCTTACCCTT ACCCTTACCCT	
	Forward *RPLP0*	CAGCAAGTGGGAAGGTGTAATCC	75pb
	Reverse *RPLP0*	CCCATTCTATCATCAACGGGTACAA	

Supplementary Table S2. Characteristics of CHEU analyzed in the study versus CHEU not selected, at randomization (day-7).

**Characteristics**	**CHEU analyzed (n = 167)**	**CHEU not selected (n = 1098)**	***p* Value[a]**
Socio-demography			
Site; n (%)			<0.01
Burkina Faso	44 (26.3)	160 (14.6)	
South Africa	39 (23.4)	181 (16.5)	
Uganda	43 (25.7)	235 (21.4)	
Zambia	41 (24.6)	522 (47.5)	
Gender; n (%)			
Boy	84 (50.3)	573 (52.2)	0.65
Anthropometry			
Weight (kg); mean ± SD	3.3 ± 0.5[†]	3.2 ± 0.5[§]	0.12
Height (cm); mean ± SD	49.6 ± 2.0	49.3 ± 2.0[ʊ]	0.06
WAZ; mean ± SD	-0.7 ± 1.3[†]	-0.5 ± 1.0[§]	0.10
HAZ; mean ± SD	-0.9 ± 1.1	-0.8 ± 1.0[ʊ]	0.05
WHZ; mean ± SD	-0.4 ± 1.3[‡]	-0.1 ± 1.2[ƍ]	0.81
Underweight (WAZ<-2); n (%)	25 (15.4)[†]	73 (6.7)[§]	0.54
Stunting (HAZ<-2); n (%)	25 (15.3)	123 (11.3)[ʊ]	0.67
Wasting (WHZ<-2); n (%)	17 (10.6)[‡]	452 (4.9)[ƍ]	0.10
Gestational age (week); median [IQR]	38.0 [38.0;40.0]	38.0 [38.0;40.0]	0.19
Preterm birth (week); n (%)			0.05
No prematurity ≥37	148 (88.6)	907 (82.6)	
Prematurity <37	19 (11.4)	191 (17.4)	
Hematology			
Hemoglobin (g/dL); mean ± SD	15.8 ± 2.1[£]	15.9 ± 2.0[§]	0.35
Hemoglobin (g/dL); n (%)			0.05
Normal >13	153 (92.2)[£]	1039 (94.6)[§]	
Anemia ≤13	13 (7.8)[£]	59 (5.4)[§]	
Mild [12;13]	10 (6.0)[£]	56 (5.1)[§]	
Moderate [10;12[	2 (1.2)[£]	2 (0.2)[§]	
Severe [9;10[	-	1 (0.1)[§]	
Very severe [0;9[	1 (0.6)[£]	-	
Platelet count ($10^3/mm^3$); n (%)			0.01
Normal ≥125	152 (93.8)[‡]	1074 (98.0)[§]	
Thrombocytopenia <125	10 (6.2)[‡]	22 (2.0)[§]	
Mild [100;125[	7 (4.3)[‡]	11 (1.0)[§]	
Moderate [50;100[	1 (0.6)[‡]	6 (0.6)[§]	
Severe [25;50[	1 (0.6)[‡]	4 (0.4)[§]	
Very severe [0;25[	1 (0.6)[‡]	1 (0.1)[§]	
White cell count ($10^3/mm^3$); n (%)			NA
Normal >2.5	166 (100.0)[£]	1097 (100.0)[§]	
Neutrophil count ($10^3/mm^3$); n (%)			
Normal >1.5	158 (96.3)[§]	1026 (94.4)[¶]	0.69

Neutropenia ≤1.5	6 (3.6)§	61 (5.6)¶
Mild [1.25;1.5]	4 (2.4)§	47 (4.3)¶
Moderate [1.0;1.25[	2 (1.2)§	12 (1.1)¶
Severe [0.75;1.0[	-	2 (0.2)¶

† one missing value, § three missing values, º ten missing values, ‡ four missing values, £ two missing values, ¶ twelve missing values. [a]Chi-square test or Fisher's exact test as appropriate and Student's t-test or Wilcoxon Mann–Whitney test for LPV/r versus 3TC. Abbreviations: SD, standard deviation; LPV/r, lopinavir/ritonavir; 3TC, lamivudine; IQR, interquartile range; WAZ, weight-for-age Z-score; HAZ, height-for-age Z-score; WHZ, weight-for-height Z-score; NA non-applicable.

Supplementary Table S3. Children's characteristics at randomization (day-7) for Burkina Faso.

Characteristics	LPV/r (n = 21)	3TC (n = 23)	Total (n = 44)	*p* Value[a]
Socio-demography				
Gender; n (%)				0.55
Boy	11 (52.4)	10 (43.5)	21 (47.7)	
Anthropometry				
Weight (kg); mean ± SD	3.0 ± 0.4†	3.1 ± 0.4	3.0 ± 0.4†	0.52
Height (cm); mean ± SD	49.9 ± 1.7	49.6 ± 1.9	49.7 ± 1.8	0.62
WAZ; mean ± SD	-1.1 ± 0.9§	-0.9 ± 1.0	-1.0 ± 1.0§	0.63
HAZ; mean ± SD	-0.4 ± 0.9	-0.6 ± 0.8	-0.5 ± 0.8	0.44
WHZ; mean ± SD	-1.2 ± 0.9§	-0.8 ± 1.1	-1.0 ± 1.0§	0.31
Underweight (WAZ<-2); n (%)	4 (21.1)§	3 (13.0)	7 (16.7)§	0.68
Stunting (HAZ<-2); n (%)	1 (4.8)	2 (8.7)	3 (6.8)	1.00
Wasting (WHZ<-2); n (%)	4 (21.1)§	2 (8.7)	6 (14.3)§	0.38
Gestational age (week); median [IQR]	39.0 [37.0;40.0]	39.0 [37.0;41.0]	39.0 [37.0;41.0]	0.59
Preterm birth (week); n (%)				1.00
No prematurity ≥37	18 (85.7)	4 (17.4)	7 (15.9)	
Prematurity <37	3 (14.3)	19 (82.6)	37 (84.1)	
Hematology				
Hemoglobin (g/dL); mean ± SD	15.3 ± 2.0	15.1 ± 2.0	15.2 ± 2.0	0.82
Hemoglobin (g/dL); n (%)				1.00
Normal >13	19 (90.5)	20 (87.0)	39 (88.6)	
Anemia ≤13	2 (9.5)	3 (13.0)	5 (11.4)	
Mild [12;13]	2 (9.5)	2 (8.7)	4 (9.1)	
Moderate [10;12[	-	1 (4.4)	1 (2.3)	
Platelet count (10^3/mm^3); n (%)				NA
Normal ≥125	21 (100.0)	23 (100.0)	44 (100.0)	
White cell count (10^3/mm^3); n (%)				NA
Normal >2.5	21 (100.0)	23 (100.0)	44 (100.0)	
Neutrophil count (10^3/mm^3); n (%)				1.00
Normal >1.5	20 (95.2)	22 (95.7)	42 (95.5)	
Neutropenia ≤1.5	1 (4.8)	1 (4.4)	2 (4.6)	
Mild [1.25;1.5]	1 (4.8)	1 (4.4)	2 (4.6)	

† one missing value, § two missing values. [a]Chi-square test or Fisher's exact test as appropriate and Wilcoxon Mann–Whitney test or Student's t-test for LPV/r versus 3TC. Abbreviations: LPV/r, lopinavir/ritonavir; 3TC, lamivudine; SD, standard deviation; WAZ, weight-for-age Z-score; HAZ, height-for-age Z-score; WHZ, weight-for-height Z-score; IQR, interquartile range; NA non-applicable.

Supplementary Table S4. Children's characteristics at randomization (day-7) for South Africa.

Characteristics	LPV/r (n = 13)	3TC (n = 26)	Total (n = 39)	*P* Value[a]
Socio-demography				
Gender; n (%)				0.65
Boy	6 (46.2)	14 (53.9)	20 (51.3)	
Anthropometry				
Weight (kg); mean ± SD	3.2 ± 0.5	3.4 ± 0.4	3.3 ± 0.5	0.53
Height (cm); mean ± SD	48.5 ± 2.1	49.4 ± 1.7	49.1 ± 1.8	0.15
WAZ; mean ± SD	0.3 ± 1.1	0.03 ± 1.0	0.1 ± 1.0	0.51
HAZ; mean ± SD	-0.7 ± 1.1	-0.8 ± 1.0†	-0.8 ± 1.0†	0.66
WHZ; mean ± SD	0.8 ± 1.0	0.5 ± 1.1†	0.6 ± 1.1†	0.52
Underweight (WAZ<-2); n (%)	-	-	-	NA
Stunting (HAZ<-2); n (%)	1 (7.7)	2 (8.0)†	3 (7.9)†	1.00
Wasting (WHZ<-2); n (%)	-	1 (4)†	1 (2.63)†	1.00
Gestational age (week); median [IQR]	38.0 [37.0;38.0]	38.0 [38.0;38.0]	38.0 [38.0;38.0]	0.28
Preterm birth (week); n (%)				
No prematurity ≥37	11 (84.6)	23 (88.5)	34 (87.2)	1.00
Prematurity <37	2 (15.4)	3 (11.5)	5 (12.8)	
Hematology				
Hemoglobin (g/dL); mean ± SD	16.6 ± 3.5	16.6 ± 2.9§	16.6 ± 3.0§	0.98
Hemoglobin (g/dL); n (%)				0.63
Normal >13	11 (84.6)	22 (91.7)§	33 (89.2)§	
Anemia ≤13	2 (15.4)	2 (8.3)§	4 (10.8)§	
Mild [12;13]	1 (7.7)	1 (4.2)§	2 (5.4)§	
Moderate [10;12[	-	1 (4.2)§	1 (2.7)§	
Very severe [0;9[	1 (7.7)	-	1 (2.7)§	
Platelet count (10^3/mm^3); n (%)				0.21
Normal ≥125	9 (69.2)	20 (90.9)‡	29 (82.9)‡	
Thrombocytopenia <125	4 (30.8)	2 (9.1) ‡	6 (17.1)‡	
Mild [100;125[	2 (15.4)	2 (9.1)‡	4 (11.4)‡	
Moderate [50;100[	1 (7.7)	-	1 (2.9)‡	
Very severe [0;25[	1 (7.7)	-	1 (2.9)‡	
White cell count (10^3/mm^3); n (%)				NA
Normal >2.5	13 (100)	24 (100.0)§	37 (100.0)§	
Neutrophil count (10^3/mm^3); n (%)				NA
Normal >2.5	13 (100)	24 (100.0)§	37 (100.0)§	

† one missing value, § two missing values, ‡ four missing values. [a]Chi-square test or Fisher's exact test as appropriate and Wilcoxon Mann–Whitney test or Student's t-test for LPV/r versus 3TC. Abbreviations: LPV/r, lopinavir/ritonavir; 3TC, lamivudine; SD, standard deviation; WAZ, weight-for-age Z-score; HAZ, height-for-age Z-score; WHZ, weight-for-height Z-score; IQR, interquartile range; NA non-applicable.

Supplementary Table S5. Children's characteristics at randomization (day-7) for Uganda.

Characteristics	LPV/r (n = 20)	3TC (n = 23)	Total (n = 43)	*p* Value[a]
Socio-demography				
Gender; n (%)				0.28
Boy	8 (40.0)	13 (56.5)	21 (48.8)	
Anthropometry				
Weight (kg); mean ± SD	3.4 ± 0.5	3.5 ± 0.5	3.5 ± 0.5	0.64
Height (cm); mean ± SD	50.0 ± 2.8	51.1 ± 1.6	50.6 ± 2.3	0.14
WAZ; mean ± SD	-0.6 ± 1.5†	-0.6 ± 1.4	0.6 ± 1.4†	0.98
HAZ; mean ± SD	-1.2 ± 1.2†	-0.7 ± 1.4	-0.9 ± 1.3†	0.21
WHZ; mean ± SD	0.04 ± 1.4†	-0.3 ± 1.2	-0.2 ± 1.3†	0.38
Underweight (WAZ<-2); n (%)	2 (10.5)†	4 (17.4)	6 (14.3)†	0.67
Stunting (HAZ<-2); n (%)	7 (36.8)†	4 (17.4)	11 (26.2)†	0.18
Wasting (WHZ<-2); n (%)	2 (10.53)†	1 (4.35)	3 (7.14)†	0.58
Gestational age (week); median [IQR]	38.0 [38.0;38.0]	38.0 [38.0;40.0]	40.0 [38.0;39.0]	0.46
Preterm birth (week); n (%)				NA
No prematurity ≥37	20 (100.0)	23 (100.0)	43 (100.0)	
Hematology				
Hemoglobin (g/dL); mean ± SD	15.8 ± 1.3	15.8 ± 1.4	15.8 ± 1.3	0.91
Hemoglobin (g/dL); n (%)				NA
Normal >13	20 (100.0)	23 (100.0)	43 (100.0)	
Platelet count (10^3/mm^3); n (%)				0.10
Normal ≥125	17 (85.0)	21 (95.5)†	38 (90.5)†	
Thrombocytopenia <125	3 (15.0)	1 (4.5)†	4 (9.5)†	
Mild [100;125[	3 (15.0)	-	3 (7.1)†	
Severe [25;50[	-	1 (4.5)†	1 (2.4)†	
White cell count (10^3/mm^3); n (%)				NA
Normal >2.5	20 (100.0)	23 (100.0)	43 (100.0)	
Neutrophil count (10^3/mm^3); n (%)				0.47
Normal >1.5	19 (95.0)	23 (100.0)	42 (97.7)	
Neutropenia ≤1.5	1 (5.0)	-	1 (2.3)	
Mild [1.25;1.5]	1 (5.0)	-	1 (2.3)	

† one missing value. [a]Chi-square test or Fisher's exact test as appropriate and Wilcoxon Mann–Whitney test or Student's t-test for LPV/r versus 3TC. Abbreviations: LPV/r, lopinavir/ritonavir; 3TC, lamivudine; SD, standard deviation; WAZ, weight-for-age Z-score; HAZ, height-for-age Z-score; WHZ, weight-for-height Z-score; IQR, interquartile range; NA non-applicable.

Supplementary Table S6. Children's characteristics at randomization (day-7) for Zambia.

**Characteristics**	**LPV/r (n = 21)**	**3TC (n = 20)**	**Total (n = 41)**	***p* Value**[a]
Socio-demography				
Gender; n (%)				0.87
Boy	11 (52.4)	11 (55.0)	22 (53.7)	
Anthropometry				
Weight (kg); mean ± SD	3.2 ± 0.4	3.2 ± 0.4	3.2 ± 0.4	0.78
Height (cm); mean ± SD	49.0 ± 1.6	48.6 ± 1.6	48.8 ± 1.6	0.49
WAZ; mean ± SD	-1.5 ± 1.1[†]	-1.0 ± 1.3[†]	-1.3 ± 1.2[§]	0.16
HAZ; mean ± SD	-1.6 ± 1.1[†]	-1.2 ± 0.9[†]	-1.4 ± 1.1[§]	0.24
WHZ; mean ± SD	-1.02 ± 1.2[†]	-0.56 ± 1.5[†]	-0.8 ± 1.3[§]	0.29
Underweight (WAZ<-2); n (%)	7 (35.0)[†]	5 (26.3)[†]	12 (30.8)[§]	0.56
Stunting (HAZ<-2); n (%)	6 (30.0)[†]	2 (10.5)[†]	8 (20.5)[§]	0.24
Wasting (WHZ<-2); n (%)	4 (20.0)[†]	3 (15.8)[†]	7 (18.0)[§]	1.00
Gestational age (week); mean ± SD	38.0 ± 1.5	38.4 ± 1.3	38.2 ± 1.4	0.42
Preterm birth (week); n (%)				0.41
No prematurity ≥37	16 (76.2)	18 (90.0)	34 (82.9)	
Prematurity <37	5 (23.8)	2 (10.0)	7 (17.1)	
Hematology				
Hemoglobin (g/dL); mean ± SD	15.6 ± 1.7	15.6 ± 2.1	15.6 ± 1.9	1.00
Hemoglobin (g/dL); n (%)				0.34
Normal >13	20 (95.2)	17 (85.0)	37 (90.2)	
Anemia ≤13	1 (4.8)	3 (15.0)	4 (9.8)	
Mild [12;13]	1 (4.8)	3 (15.0)	4 (9.8)	
Platelet count (10^3/mm^3); n (%)				NA
Normal ≥125	21 (100.0)	20 (100.0)	41 (100.0)	
White cell count (10^3/mm^3); n (%)				NA
Normal >2.5	21 (100.0)	20 (100.0)	41 (100.0)	
Neutrophil count (10^3/mm^3); n (%)				0.49
Normal >1.5	19 (90.5)	18 (94.7)[†]	37 (92.5)[†]	
Neutropenia ≤1.5	2 (9.5)	1 (5.3)[†]	3 (7.5)[†]	
Mild [1.25;1.5]	-	1 (5.3)[†]	1 (2.5)[†]	
Moderate [1.0;1.25[	2 (9.5)	-	2 (5.0)[†]	

[†] one missing value, [§] two missing values. [a]Chi square test or Fisher's exact test as appropriate and Student's t-test for LPV/r versus 3TC. Abbreviations: LPV/r, lopinavir/ritonavir; 3TC, lamivudine; SD, standard deviation; WAZ, weight-for-age Z-score; HAZ, height-for-age Z-score; WHZ, weight-for-height Z-score; IQR, interquartile range; NA non-applicable.

Supplementary Table S7. Maternal characteristics at randomization (day-7) and during follow up of the PROMISE PEP trial for Burkina Faso.

**Characteristics**	**LPV/r (n = 21)**	**3TC (n = 23)**	**Total (n = 44)**	***p* Value**[a]
**At randomization (day-7)**				
Socio-demography				
Age (year); median [IQR]	31.2 [27.3;35.3]	29.5 [20.9;32.6]	30.3 [25.1;33.7]	0.06
Parity; median [IQR]	3.0 [3.0;4.0]	2.0 [1.4;4.0]	3.0 [1.5;4.0]	0.09
Children under 5 years living in the household; n (%)				0.12
0	14 (66.7)	11 (47.8)	25 (56.8)	
1	4 (19.1)	11 (47.8)	15 (34.1)	
2	2 (9.5)	1 (4.4)	3 (6.8)	
3	1 (4.8)	-	1 (2.3)	
Education				
Mother/caregiver ever attended school; n (%)				0.07
Yes	9 (42.9)	16 (69.6)	25 (56.8)	
Economic status				
Type of income; n (%)				0.75
No income generating activities	3 (14.3)	2 (8.7)	5 (11.4)	
Irregular income	13 (61.9)	13 (56.5)	26 (59.1)	
Regular income	5 (23.8)	8 (34.8)	13 (29.6)	
Clinical and biological characteristics				
BMI; median [IQR]	23.9 [21.2;26.1]	22.7 [21.4;24.4]	22.7 [21.2;25.9]	0.98
CD4 cells count (cells/mm^3); median [IQR]	507.0 [411.0;552.0]	502.0 [425.0;632.0]	504 [418.0;598.0]	0.46
HIV viral load control; n (%)				0.04
<1000 copies/mL	8 (38.1)	16 (69.6)	24 (54.5)	
≥1000 copies/mL	13 (61.9)	7 (30.4)	20 (45.5)	
WHO HIV staging; n (%)				0.60
Stage 1	19 (90.5)	22 (95.7)	41 (93.2)	
Stage 2	2 (9.5)	1 (4.3)	3 (6.8)	

Maternal prophylaxis during pregnancy				
ARV regimen; n (%)				1.00
ZDV	21 (100)	22 (95.7)	43 (97.7)	
No ARV	-	1 (4.3)	1 (2.3)	
Duration of ARV prophylaxis taken during pregnancy (week); median [IQR]	9.0 [8.0;12.0]	9.0 [5.0;11.0]	9.0 [6.0;11.0]	0.37
**During the PROMISE PEP trial**				
HIV viral load at week-38 (Log copies/mL); median [IQR]	4.8 [4.0;5.4]†	4.6 [3.4;4.9]§	4.7 [4.0;5.2]‡	0.44
HIV viral load control at week-38; n (%)				0.35
<1000 copies/mL	3 (16.7)†	1 (6.3)§	4 (11.8)‡	
≥1000 copies/mL	15 (83.3)†	15 (93.8)§	30 (88.2)‡	
Duration of breastfeeding (week); median [IQR]	46.6 [45.1;48.3]	47.7 [45.71;50.0]	47.0 [45.2;49.8]	0.20
Smoking during pregnancy; n (%)				NA
No	20 (100.0)£	21 (100.0)º	41 (100.0)†	
Smoking during breastfeeding; n (%)				NA
No	20 (100.0)£	21 (100.0)º	41 (100.0)†	
Alcohol consumption during pregnancy; n (%)				0.88
Yes	10 (50)£	10 (47.62)º	20 (48.78)†	

† three missing values, § seven missing values, ‡ ten missing values, £ one missing value, º two missing values. [a]Chi-square test or Fisher's exact test as appropriate and Wilcoxon Mann–Whitney test for LPV/r versus 3TC. Abbreviations: LPV/r, lopinavir/ritonavir; 3TC, lamivudine; IQR, interquartile range; BMI, body mass index; HIV, human immunodeficiency virus; WHO, World Health Organization; ARV, antiretroviral; ZDV, zidovudine.

Supplementary Table S8. Maternal characteristics at randomization (day-7) and during follow up of the PROMISE PEP trial for South Africa.

Characteristics	LPV/r (n = 13)	3TC (n = 26)	Total (n = 39)	*p* Value[a]
**At randomization (day-7)**				
Socio-demography				
Age (year); median [IQR]	27.7 [27.0;33.8]	30.4 [25.7;34.7]	29.7 [25.7;34.4]	0.85
Parity; median [IQR]	2.0 [1.0;3.0]	2.0 [1.0;3.0]	2.0 [1.0;3.0]	0.58
Children under 5 years living in the household; n (%)				0.37
0	4 (50.0)[†]	13 (76.5)[§]	17 (68.0)[‡]	
1	2 (25.0)[†]	3 (17.7)[§]	5 (20.0)[‡]	
2	2 (25.0)[†]	1 (5.9)[§]	3 (12.0)[‡]	
Education				
Mother/caregiver ever attended school; n (%)				NA
Yes	8 (100.0)[†]	17 (100.0)[§]	25 (100.0)[‡]	
Economic status				
Type of income; n (%)				0.16
Irregular income	4 (50.0)[†]	3 (17.6)[§]	7 (28.0)[‡]	
Regular income	4 (50.0)[†]	14 (82.4)[§]	18 (72.0)[‡]	
Clinical and biological characteristics				
BMI; median [IQR]	27.0 [24.2;28.0]	28.4 [23.2;33.7]	27.9 [23.9;32.3]	0.51
CD4 cells count (cells/mm^3); median [IQR]	504.0 [433.0;579.0]	455.5 [424.0;534.0]	458.0 [424.0;566.0]	0.34
HIV viral load control; n (%)				0.49
<1000 copies/mL	9 (69.2)	15 (57.7)	24 (61.5)	
≥1000 copies/mL	4 (30.8)	11 (42.3)	15 (38.5)	
WHO HIV staging; n (%)				NA
Stage 1	13 (100.0)	26 (100.0)	39 (100.0)	
Maternal prophylaxis during pregnancy				
ARV regimen; n (%)				NA
ZDV	13 (100.0)	26 (100.0)	39 (100.0)	

Duration of ARV prophylaxis taken during pregnancy (week); median [IQR]	11.0 [8.0;16.0]	14.0 [8.0;16.0]	12.0 [8.0;16.0]	0.98
**During the PROMISE PEP trial**				
HIV viral load at week-38 (Log copies/mL); median [IQR]	3.1 [2.4;3.5][£]	3.9 [2.9;4.5][º]	3.5 [2.8;4.2][†]	0.04
HIV viral load control at week-38; n (%)				0.71
<1000 copies/mL	5 (41.7)[£]	7 (31.8)[º]	12 (35.3)[†]	
≥1000 copies/mL	7 (58.3)[£]	15 (68.2)[º]	22 (64.7)[†]	
Duration of breastfeeding (week); median [IQR]	29.6 [11.7;45.3]	46.3 [34.0;49.9]	43.3 [30.3;49.0]	0.02
Smoking during pregnancy; n (%)				1.00
No	6 (85.7)[♀]	13 (86.7)[¶]	19 (86.4)[ł]	
Smoking during breastfeeding; n (%)				1.00
No	6 (85.7)[♀]	14 (93.3)[¶]	20 (90.9)[ł]	
Alcohol consumption during pregnancy; n (%)				1.00
Yes	3 (20.0)[♀]	2 (28.6)[¶]	5 (22.7)[ł]	

[†] five missing values, [§] nine missing values, [‡] fourteen missing values, [£] one missing value, [º] four missing values, [♀] six missing values, [¶] eleven missing values, [ł] seventeen missing values. [a]Chi-square test or Fisher's exact test as appropriate and Wilcoxon Mann–Whitney test for LPV/r versus 3TC. Abbreviations: LPV/r, lopinavir/ritonavir; 3TC, lamivudine; IQR, interquartile range; BMI, body mass index; HIV, human immunodeficiency virus; WHO, World Health Organization; ARV, antiretroviral; ZDV, zidovudine.

Supplementary Table S9. Maternal characteristics at randomization (day-7) and during follow up of the PROMISE PEP trial for Uganda.

**Characteristics**	**LPV/r (n = 20)**	**3TC (n = 23)**	**Total (n = 43)**	***p* Value**[a]
**At randomization (day-7)**				
Socio-demography				
Age (year); median [IQR]	28.6 [24.4;31.0]	28.3 [24.2;30.2]	28.3 [24.2 30.2]	0.98
Parity; median [IQR]	3.5 [3.0;5.0]	3.0 [2.0;5.0]	3.0 [2.0;5.0]	0.74
Children under 5 years living in the household; n (%)				0.58
0	8 (40.0)	6 (26.1)	14 (32.6)	
1	7 (35.0)	13 (56.2)	20 (46.5)	
2	4 (20.0)	3 (13.0)	7 (16.3)	
3	1 (5.0)	1 (4.4)	2 (4.7)	
Education				
Mother/caregiver ever attended school; n (%)				1.00
Yes	21 (91.3)	18 (90.0)	39 (90.7)	
Economic status				
Type of income; n (%)				0.91
No income generating activities	11 (55.0)	11 (47.8)	22 (51.2)	
Irregular income	3 (15.0)	3 (13.0)	6 (14.0)	
Regular income	6 (30.0)	9 (39.1)	15 (34.9)	
Clinical and biological characteristics				
BMI; median [IQR]	22.9 [21.8;24.5]	22.7 [20.2;24.2]	22.8 [21.1;24.4]	0.30
CD4 cells count (cells/mm^3); median [IQR]	513.5 [451.5;642.0]	530.0 [439.0;576.0]	523.0 [443.0;601.0]	0.79
HIV viral load control; n (%)				0.72
<1000 copies/mL	16 (80.0)	16 (72.7)[†]	32 (76.2)[†]	
≥1000 copies/mL	4 (20.0)	6 (27.3)[†]	10 (23.8)[†]	
WHO HIV staging; n (%)				0.24
Stage 1	20 (100.0)	20 (87.0)	40 (93.0)	
Stage 2	-	3 (13.04	3 (7.0)	

Maternal prophylaxis during pregnancy				
ARV regimen; n (%)				0.11
ZDV	10 (50.0)	11 (47.8)	21 (48.8)	
AZT+3TC	5 (25.0)	11 (47.8)	16 (37.2)	
None	5 (25.0)	1 (4.3)	6 (14.0)	
Duration of ARV prophylaxis taken during pregnancy (week); median [IQR]	11.0 [6.0;12.0]§	6.5 [4.0;10.0]†	8.0 [4.0;12.0]‡	0.10
**During the PROMISE PEP trial**				
HIV viral load at week-38 (Log copies/mL); median [IQR]	2.6 [2.2;3.9]£	2.2 [2.2;3.9]†	2.2 [2.2;3.9]§	0.57
HIV viral load control at week-38; n (%)				0.26
<1000 copies/mL	9 (56.3)£	14 (63.6)†	23 (60.5)§	
≥1000 copies/mL	7 (43.8)£	8 (33.4)†	15 (39.5)§	
Duration of breastfeeding (week); median [IQR]	42.3 [37.4;45.4]†	38.9 [33.4;42.4]	40.3 [35.3;43.6]	0.28
Smoking during pregnancy; n (%)				NA
No	16 (100.0)£	21 (100.0)º	37 (100.0)‡	
Smoking during breastfeeding; n (%)				NA
No	16 (100.0)£	21 (100.0)º	37 (100.0)‡	
Alcohol consumption during pregnancy; n (%)				0.67
Yes	5 (31.3)£	8 (38.1)º	13 (35.1)‡	

† one missing value, § five missing values, ‡ six missing values, £ four missing value, º two missing values. [a]Chi-square test or Fisher's exact test as appropriate and Wilcoxon Mann–Whitney test for LPV/r versus 3TC. Abbreviations: LPV/r, lopinavir/ritonavir; 3TC, lamivudine; IQR, interquartile range; BMI, body mass index; HIV, human immunodeficiency virus; WHO, World Health Organization; ARV, antiretroviral; ZDV, zidovudine.

Supplementary Table S10. Maternal characteristics at randomization (day-7) and during follow up of the PROMISE PEP trial for Zambia.

**Characteristics**	**LPV/r (n = 21)**	**3TC (n = 20)**	**Total (n = 41)**	***p* Value[a]**
**At randomization (day-7)**				
Socio-demography				
Age (year); median [IQR]	28.8 [25.5;32.5]	27.9 [25.7;34.5]	28.7 [25.6;33.6]	0.67
Parity; median [IQR]	3.0 [2.0;4.0]	3.0 [3.0;4.0]	3.0 [2.0;4.0]	0.11
Children under 5 years living in the household; n (%)				0.02
0	15 (71.4)	7 (35.0)	22 (53.7)	
1	4 (19.1)	12 (60.0)	16 (39.0)	
2	2 (9.5)	1 (5.0)	3 (7.3)	
Education				
Mother/caregiver ever attended school; n (%)				NA
Yes	21 (100.0)	20 (100.0)	41 (100.0)	
Economic status				
Type of income; n (%)				1.00
No income generating activities	1 (4.8)	-	1 (2.4)	
Irregular income	16 (76.2)	15 (75.0)	31 (75.6)	
Regular income	4 (19.0)	5 (25.0)	9 (22.0)	
Clinical and biological characteristics				
BMI; median [IQR]	22.7 [21.6;25.2]	25.3 [23.23;28.0]	24.5 [21.7;26.5]	0.15
CD4 cells count (cells/mm^3); median [IQR]	467.0 [408.0;658.0]	484.5 [419.5;683.5]	477.0 [408.0;667.5]	0.81
HIV viral load control; n (%)				0.65
<1000 copies/mL	9 (42.9)	10 (50.0)	19 (46.3)	
≥1000 copies/mL	12 (57.1)	10 (50.0)	22 (53.7)	
WHO HIV staging; n (%)				NA
Stage 1	21 (100.0)	20 (100.0)	41 (100.0)	
Maternal prophylaxis during pregnancy				
ARV regimen; n (%)				NA

ZDV	21 (100.0)	20 (100.0)	41 (100.0)	
Duration of ARV prophylaxis taken during pregnancy (week); median [IQR]	8.0 [5.0;10.0]	8.0 [5.0;9.5]	8.0 [5.0;10.0]	0.51
**During the PROMISE PEP trial**				
HIV viral load at week-38 (Log copies/mL); median [IQR]	5.0 [3.6;5.2]†	4.4 [3.8;5.2]§	4.7 [3.7;5.2]‡	0.81
HIV viral load control at week-38; n (%)				0.45
<1000 copies/mL	3 (15.0)†	2 (12.5)§	5 (13.9)‡	
≥1000 copies/mL	17 (85.0)†	14 (87.5)§	31 (86.1)‡	
Duration of breastfeeding (week); median [IQR]	38.7 [37.0;44.1]	35.1 [32.1;41.3]	38.5 [33.7;41.6]	0.09
Smoking during pregnancy; n (%)				NA
No	20 (100.0)†	18 (100.0)£	38 (100.0)º	
Smoking during breastfeeding; n (%)				NA
No	20 (100.0)†	18 (100.0)£	38 (100.0)º	
Alcohol consumption during pregnancy; n (%)				0.39
Yes	7 (35.0)†	4 (22.2)£	11 (29.0)º	

† one missing value, § four missing values, ‡ five missing values, £ two missing value, º three missing values. [a]Chi-square test or Fisher's exact test as appropriate and Wilcoxon Mann–Whitney test for LPV/r versus 3TC. Abbreviations: LPV/r, lopinavir/ritonavir; 3TC, lamivudine; IQR, interquartile range; BMI, body mass index; HIV, human immunodeficiency virus; WHO, World Health Organization; ARV, antiretroviral; ZDV, zidovudine.

Supplementary Table S11. Children' telomere length at day-7 according to gender.

Site	PrEP	n	Female median [IQR]	n	Male median [IQR]	*p* Value[a]
Burkina Faso	Any	23	331 [131;459]	21	355 [214;467]	0.71
	LPV/r	10	262 [131;403]	11	355 [95;562]	0.60
	3TC	13	360 [307;482]	10	372 [231;459]	0.65
South Africa	Any	19	131 [109;147]	20	132 [119;162]	0.37
	LPV/r	7	117 [70;144]	6	125 [110;165]	0.20
	3TC	12	131 [112;147]	14	132 [116;147]	0.90
Uganda	Any	22	436 [332;485]	21	308 [268;410]	0.21
	LPV/r	12	473 [350;529]	8	331 [231;487]	0.28
	3TC	10	413 [226;447]	13	308 [276;410]	0.69
Zambia	Any	19	390 [368;485]	22	324 [208;367]	0.12
	LPV/r	10	330 [295;485]	11	331 [275;402]	0.70
	3TC	9	458 [268;464]	11	305 [105;346]	0.19
All	Any	83	322 [132;459]	84	272 [147;397]	0.33
	LPV/r	39	322 [178;483]	36	298 [150;407]	0.68
	3TC	44	319 [128;454]	48	258 [144;417]	0.52

[a]Wilcoxon Mann–Whitney test for Female versus Male. Abbreviations: PrEP, pre-exposure prophylaxis; LPV/r, lopinavir/ritonavir; 3TC, lamivudine; IQR, interquartile range.

Supplementary Table S12. Children's characteristics at week-50 .

**Characteristics**	**LPV/r (n = 75)**	**3TC (n = 92)**	**Total (n = 167)**	***p* Value**[a]
Socio-demography				
Site; n (%)				0.40
Burkina Faso	21 (28.0)	23 (25.0)	44 (26.4)	
South Africa	13 (17.3)	26 (28.3)	39 (23.4)	
Uganda	20 (26.7)	23 (25.0)	43 (25.7)	
Zambia	21 (28.0)	20 (21.7)	41 (24.6)	
Gender; n (%)				
Boy	36 (48.0)	48 (52.2)	84 (50.3)	0.59
Anthropometry				
Weight (kg); mean ± SD	8.5 ± 1.4[†]	8.8 ± 1.2[§]	8.7 ± 1.3[‡]	0.14
Height (cm); mean ± SD	72.1 ± 3.1[£]	72.8 ± 2.2[£]	72.5 ± 2.9[‡]	0.13
WAZ; mean ± SD	-0.8 ± 1.3[‡]	-0.6 ± 1.2[§]	-0.7 ± 1.3[ʊ]	0.21
HAZ; mean ± SD	-1.0 ± 1.1[£]	-0.8 ± 1.1[£]	-0.9 ± 1.1[‡]	0.32
WHZ; mean ± SD	-0.5 ± 1.4[‡]	-0.3 ± 1.3[£]	-0.4 ± 1.3[ϱ]	0.40
Underweight (WAZ<-2); n (%)	13 (18.3) 3[‡]	12 (13.2)[§]	25 (15.4)[ʊ]	0.37
Stunting (HAZ<-2); n (%)	15 (20.6)[£]	10 (11.1)[£]	25 (15.3)[‡]	0.10
Wasting (WHZ<-2); n (%)	10 (14.1)[‡]	7 (7.8)[£]	17 (10.6)[ϱ]	0.20
Gestational age (week); median [IQR]	38.0 [38.0;40.0]	38.0 [38.0;40.0]	38.0 [38.0;40.0]	0.37
Preterm birth (week); n (%)				0.47
No prematurity ≥37	65 (86.7)	83 (90.2)	148 (88.6)	
Prematurity <37	10 (13.3)	9 (9.8)	19 (11.4)	
Hematology				
Hemoglobin (g/dL); mean ± SD	10.2 ± 1.3[¤]	10.4 ± 1.1[¥]	10.3 ± 1.2[Σ]	0.27
Hemoglobin (g/dL); n (%)				0.61
Normal >10.9	15 (22.4%)	25 (30.9%)	40 (27.0%)	
Anemia ≤10.9	52 (77.6%)	56 (69.1%)	108 (73.0%)	
Mild 10.0;10.9]	24 (35.8%)	29 (35.8%)	53 (35.8%)	
Moderate [9.0;10.9[	19 (28.4%)	19 (23.5%)	38 (25.7%)	
Severe [7.0;9.0[	8 (11.9%)	8 (9.9%)	16 (10.8%)	
Very severe <7.0	1 (1.5%)	-	1 (0.7%)	
Platelet count (10^3/mm^3); n (%)				0.27
Normal ≥125	66 (98.5)[¤]	79 (97.5)[¥]	145 (98.0)[Σ]	
Thrombocytopenia <125	1 (1.5)[¤]	2 (2.5)[¥]	3 (2.0)[Σ]	
Mild [100;125[	1 (1.5)[¤]	1 (1.2)[¥]	2 (1.4)[Σ]	
Moderate [50;100[	-	1 (1.2)[¥]	1 (0.7)[Σ]	
White cell count (10^3/mm^3); n (%)				NA
Normal >2.5	67 (100.0)[¤]	81 (100.0)[¥]	148 (100.0)[Σ]	
Neutrophil count (10^3/mm^3); n (%)				0.28
Normal >1.3	54 (80.6%)[¤]	67 (82.7%)	121 (81.8%)	
Neutropenia ≤1.3	13 (19.4%)[¤]	14 (17.3%)	27 (18.2%)	
Mild [1.0;1.3]	7 (10.5%)[¤]	9 (11.1%)[¥]	16 (10.8%)[Σ]	

Moderate [0.75;1.0[	5 (7.5%)[¤]	1 (1.2%)[¥]	6 (4.1%)[Σ]
Severe [0.5;0.75[	1 (1.5%)[¤]	3 (3.7%)[¥]	4 (2.7%)[Σ]
Very severe <0.5	-	1 (1.2%)[¥]	1 (0.7%)[Σ]

[†] three missing values, [§] one missing value, [‡] four missing values, [£] two missing values, [º] five missing values, ϱ six missing values, ¤ eight missing values, ¥ eleven missing values, Σ nineteen missing values. [a]Chi-square test or Fisher's exact test as appropriate and Student's t-test or Wilcoxon Mann–Whitney test for LPV/r versus 3TC. Abbreviations: SD, standard deviation; LPV/r, lopinavir/ritonavir; 3TC, lamivudine; IQR, interquartile range; WAZ, weight-for-age Z-score, HAZ, height-for-age Z-score; WHZ, weight-for-height Z-score, NA non-applicable.

Supplementary Table S13. Characteristics of enrolled CHEU aged six years from the PROMISE M&S trial.

**Characteristic**	**LPV/r (n = 62)**	**3TC (n = 66)**	**Total (n = 128)**	***p* Value[a]**
Socio-demography				
Age (year); mean [IQR]	5.6 ± 0.6	5.7 ± 0.6	5.7 ± 0.6	0.21
Site; n (%)				0.90
Burkina Faso	21 (33.9)	23 (34.9)	44 (34.4)	
Uganda	20 (32.3)	23 (34.9)	43 (33.6)	
Zambia	21 (33.9)	20 (30.3)	41 (32.0)	
Gender; n (%)				
Male	29 (46.8)	33 (50.0)	62 (48.4)	0.72
Anthropometry				
Weight (kg); mean ± SD	19.3 ± 2.3[†]	19.5 ± 2.9	19.4 ±2.6[†]	0.59
Height (cm); mean ± SD	114.5 ± 5.1[†]	114.6 ± 6.4	114.6 ± 5.8[†]	0.93
WAZ; mean ± SD	-0.6 ± 0.7[†]	-0.6 ± 0.9	-0.6 ± 0.8[†]	0.85
HAZ; mean ± SD	-0.4 ± 0.8[†]	-0.5 ± 1.1	-0.4 ± 1.0[†]	0.58
BMIZ; mean ± SD	-0.5 ± 0.9[†]	-0.4 ± 0.8	-0.5 ± 0.8[†]	0.50
Hematology; n (%)				
Hemoglobin concentration (g/dL)				0.85
Normal >10.4	58 (93.6)	62 (93.9)	120 (93.8)	
Anemia ≤10.4	4 (6.5)	4 (6.1)	8 (6.3)	
Mild [10.4-9.5[	4 (6.5)	3 (4.6)	7 (5.5)	
Moderate [9.5-8.5[	-	1 (1.5)	1 (0.8)	
Platelet count ($10^3/mm^3$)				NA
Normal >125	62 (100.0)	66 (100.0)	128 (100.0)	
Leucocyte count ($10^3/mm^3$)				NA
Normal >2.5	62 (100.0)	66 (100.0)	128 (100.0)	
Neutrophil count ($10^3/mm^3$)				0.24
Normal >1.0	60 (93.8)	65 (100.0)[†]	125 (98.4)[†]	
Neutropenia ≤1.0	2 (3.2)	-	2 (1.6)[†]	
Mild [1.0-0.79[	2 (3.2)	-	2 (1.6)[†]	
Medical events; n (%)				
Clinical consultation without admission during the last year				0.15
Yes	46 (75.4)[†]	42 (63.6)	88 (69.3)[†]	
Hospital admission since week-50				0.78
Yes	18 (30.0)[§]	18 (27.7)[†]	36 (28.8)[‡]	
Neuropsychological assessment				
SDQ-25; median [IQR]	7.0 [3.0;10.0][†]	5.0 [4.0;10.0]	6.0 [3.0;10.0][†]	0.53
TOVA; mean ± SD	2.1 ± 0.7	2.2 ±0.8	2.2 ± 0.8[b]	0.53

MABC-2; mean ± SD	78.5 ± 11.0	77.0 ± 10.5	77.7 ± 10.8[b]	0.45
KABC-II; mean ± SD	52.1 ± 12.3	51.6 ± 16.0	51.9 ± 14.3[b]	0.82

† one missing value, § two missing values, ‡ three missing values. [a]Chi-square test or Fisher's exact test as appropriate and Wilcoxon Mann–Whitney test or Student's t-test for LPV/r versus 3TC. [b]For TOVA test, 119 CHEU had valid test (54 for LPV/r and 65 for 3TC); for MABC-2, 126 CHEU had valid test (60 for LPV/r and 66 for 3TC) and for KABC-II, 126 CHEU had valid test (61 for LPV/r and 65 for 3TC). Abbreviations: CHEU, children who are HIV-exposed uninfected; IQR, interquartile range; SD, standard deviation; WAZ, weight-for-age Z-score; HAZ, height-for-age Z-score; BMIZ, body mass index Z-score; NA, non-applicable; ARV, antiretroviral; SDQ-25, strength and difficulties questionnaire; TOVA, test of variable of attention; MABC-2, movement assessment battery for children second edition; KABC-II, Kaufman assessment battery for children second edition.

Supplementary Table S14. Characteristics at week-50 of CHEU with or without telomere shortening at week-50.

Characteristics	CHEU without telomere shortening (n = 71)	CHEU with telomere shortening (n = 57)	Total (n = 128)	*p* Value[a]
Socio-demography				
Site; n (%)				0.09
Burkina Faso	27 (38.0%)	17 (29.8%)	44 (34.4%)	
Uganda	18 (25.4%)	25 (43.9%)	43 (33.6%)	
Zambia	26 (36.6%)	15 (26.3%)	41 (32.0%)	
Gender; n (%)				
Boy	37 (52.1)	27 (47.4)	64 (50.0)	0.59
Anthropometry				
Weight (kg); mean ± SD	8.4 ± 1.1[†]	8.5 ±1.4[†]	8.4 ± 1.2[§]	0.65
Height (cm); mean ± SD	72.4 ± 2.7[†]	72.3 ± 3.2[‡]	72.4 ± 2.9[£]	0.81
WAZ; mean ± SD	-0.96 ± 1.11[†]	-0.91±1.39[£]	-0.94 ± 1.23[ʊ]	0.83
HAZ; mean ± SD	-0.87 ± 1.07[†]	-0.99 ±1.15[‡]	-0.92 ±1.10[£]	0.58
WHZ; mean ± SD	-0.71 ± 1.07[†]	-0.57 ±1.48[£]	-0.64 ±1.27[ʊ]	0.56
Underweight (WAZ<-2); n (%)	14 (20.3%)[†]	11 (20.4%)[£]	25 (20.3%)[ʊ]	0.99
Stunting (HAZ<-2); n (%)	10 (14.5%)[†]	10 (17.9%)[‡]	20 (16.0%)[£]	0.61
Wasting (WHZ<-2); n (%)	7 (10.1%)[†]	9 (16.7%)[£]	16 (13.0%)[ʊ]	0.29
Gestational age (week); median [IQR]	38.0 [38.0-40.0]	40.0 [38.0-40.0]	39.0 [38.0-40.0]	<0.01
Preterm birth (week); n (%)				0.20
No prematurity ≥37	61 (85.9%)	53 (93.0%)	114 (89.1%)	
Prematurity <37	10 (14.1%)	4 (7.0%)	14 (10.9%)	
Hematology				
Hemoglobin (g/dL); mean ± SD	10.0 ±1.1[♀]	10.0 ± 1.1[¥]	10.0 ±1.1[¤]	0.70
Hemoglobin (g/dL); n (%)				0.92
Normal >10.9	12 (20.0%)[♀]	9 (18.0%)[¥]	21 (19.1%)[¤]	
Anemia ≤10.9	48 (80.0%)[♀]	41 (82.0%)[¥]	89 (80.9%)[¤]	
Mild 10.0;10.9]	21 (35.0%)[♀]	18 (36.0%)[¥]	39 (35.5%)[¤]	
Moderate [9.0;10.9[	18 (30.0%)[♀]	16 (32.0%)[¥]	34 (30.9%)[¤]	
Severe [7.0;9.0[	8 (13.3%)[♀]	7 (14.0%)[¥]	15 (13.6%)[¤]	
Very severe <7.0	1 (1.7%)[♀]	-	1 (0.9%)[¤]	
Platelet count (10^3/mm^3); n (%)				0.73
Normal ≥125	59 (98.3%)[♀]	48 (96%)[¥]	107 (97.3%)[¤]	
Thrombocytopenia <125	1 (1.7%)[♀]	2 (4.0%)[¥]	3 (2.7%)[¤]	
Mild [100;125[	1 (1.7%)[♀]	1 (2.0%)[¥]	2 (1.8%)[¤]	
Moderate [50;100[	-	1 (2.0%)[¥]	1 (0.9%)[¤]	
White cell count (10^3/mm^3); n (%)				NA
Normal >2.5	60 (100.0%)[♀]	50 (100.0%)[¥]	110 (100.0%)[¤]	
Neutrophil count (10^3/mm^3); n (%)				0.73
Normal >1.3	48 (80.0%)[♀]	39 (78.0%)[¥]	87 (79.1%)[¤]	
Neutropenia ≤1.3	12 (20.0%)[♀]	11 (22.0%)[¥]	23 (20.9%)[¤]	

Mild [1.0;1.3]	8 (13.3%)[ϱ]	6 (12.0%)[¥]	14 (12.7%)[¤]	
Moderate [0.75;1.0[	2 (3.3%)[ϱ]	4 (8.0%)[¥]	6 (5.5%)[¤]	
Severe [0.5;0.75[	2 (3.3%)[ϱ]	1 (2.0%)[¥]	3 (2.7%)[¤]	
Other				
Antiretroviral prophylaxis				0.62
Lamivudine	38 (46.5)	28 (49.1)	66 (51.6)	
Lopinavir/ritonavir	33 (46.5)	29 (50.9)	62 (48.4)	

[†] two missing values, [§] four missing values, [‡] one missing value, [£] three missing values, [ʊ] five missing values, ϱ eleven missing values, [¥] seven missing values, [¤] eighteen missing values. [a] Chi-square test or Fisher's exact test as appropriate and Student's t-test or Wilcoxon Mann–Whitney test for "CHEU with no TS" versus "CHEU with TS". Abbreviations: CHEU, children who are HIV-exposed uninfected; SD, standard deviation; WAZ, weight-for-age Z-score; HAZ, height-for-age Z-score; WHZ, weight-for-height Z-score; IQR, interquartile range; NA, non-applicable.
